# Neither amphetamine nor sub-anesthetic ketamine treatment during adolescence impairs devaluation in rats tested during adulthood

**DOI:** 10.31083/j.jin2304083

**Published:** 2024-04-18

**Authors:** Ian R. Davis, Hayley Fisher, Caitlin McLean, Jackson Murray, Charles L. Pickens

**Affiliations:** 1 Department of Psychological Sciences, Kansas State University, Manhattan, KS, 66506, USA

**Keywords:** amphetamine, ketamine, devaluation, decision-making, addiction, schizophrenia

## Abstract

**Background:**

Much of the existing animal literature on the devaluation task suggests that prior repeated exposure to drugs of abuse during adulthood can impair goal-directed action, but the literature on human drug users is mixed. Also, the initiation of drug use often occurs during adolescence, but examinations of the effects of drug exposure during adolescence on behavior in the devaluation task are lacking.

**Methods:**

We examined whether repeated exposure during adolescence to amphetamine (3 mg/kg injections every-other day from post-natal day 27–45) or ketamine (twice daily 30 mg/kg injections from post-natal day 35–44) would impair behavior in a devaluation test when tested drug-free in adulthood. Rats were trained to press a left lever with a steady cue-light above it for one reinforcer and a right lever with a flashing cue-light above it for a different reinforcer. We tested whether any impairments in goal-directed action could be overcome by compensation between strategies by giving rats information based on lever-location and cue-lights during the test that was either congruent (allowing compensation) or incongruent (preventing compensation between strategies) with the configurations during training.

**Results:**

Our results provided no evidence for impairment of goal-directed action during adulthood after adolescent amphetamine or ketamine exposure.

**Conclusions:**

We discuss possible reasons for this discrepancy with the prior literature, including 1) the age of exposure and 2) the pattern in the previous literature that most previous demonstrations of drug exposure impairing devaluation in laboratory animals may be attributed to either drug-associated cues present in the testing environment and/or accelerated habit learning in tasks that predispose laboratory animals towards habit formation with extended training (with training procedures that should resist the formation of habits in the current experiment). However, additional research is needed to examine the effects of these factors, as well a potential role for the particular doses and washout periods to determine the cause of our finding of no devaluation impairment after drug exposure.

## 1. Introduction

Goals can change over time, and it is important to adapt our behavior as our goals change (goal-directed behavior). We also need to integrate information from several past experiences to guide our behavior. One task often used to model goal-directed behavior is the devaluation task [1, 2]. In operant devaluation tasks, specific responses earn specific rewards/reinforcers. The outcome (e.g., food) value is then decreased. In a third stage, the devaluation effect is measured (in extinction) by determining if there is a reduction in responses that previously earned the outcome.

Previous research has found that the ability to decrease responses to devalued rewards is impaired in multiple psychiatric disorders [3–7] (but see [8]), but the evidence for impaired goal-directed behavior in human drug users is mixed [9–13]. This mixed evidence in the literature is observed even though addictive drug use is associated with abnormalities in many human brain areas associated with flexible behavior that is goal-directed, including orbitofrontal cortex (OFC), amygdala, dorsal and ventral striatum, and prefrontal cortex areas analogous to the rodent prelimbic cortex (PL) [14–19]. The literature on goal-directed behavior impairments in human drug users may be mixed due to compensatory mechanisms [20–24], in which functions subserved by impaired brain regions are assumed by other brain regions. If the neural substrates of the strategy that usually guide goal-directed behavior were dysfunctional, apparently normal behavior could be maintained through behavioral compensation. Behavioral compensation occurs when function is maintained after a neurobiological disruption through use of an alternative strategy. However, there is minimal research on potential compensatory changes in the devaluation task because devaluation researchers design experiments to isolate the particular associations guiding goal-directed behavior. Specifically, devaluation tasks often provide information about which responses will earn specific outcomes either through information about a fixed spatial location of the response *or* through discrete stimuli that can be presented for short periods of time (such as lights, tones or objects/pictures on a touchscreen), but rarely with both types of information within the same task. There are several sets of terminology that could be used for this distinction, and it is possible to think of the spatial location as a “location cue” in which case both sources of information represent different types of cues, but we will largely refer to use of the different types of information as different “strategies”. Separation of tasks into those that can only be guided by responses to a location [25–29] or can only be guided by discrete stimuli [30–39] prevents study of how a new strategy might be used when the neural substrates of the usual strategy are compromised.

**Fig. 1.**
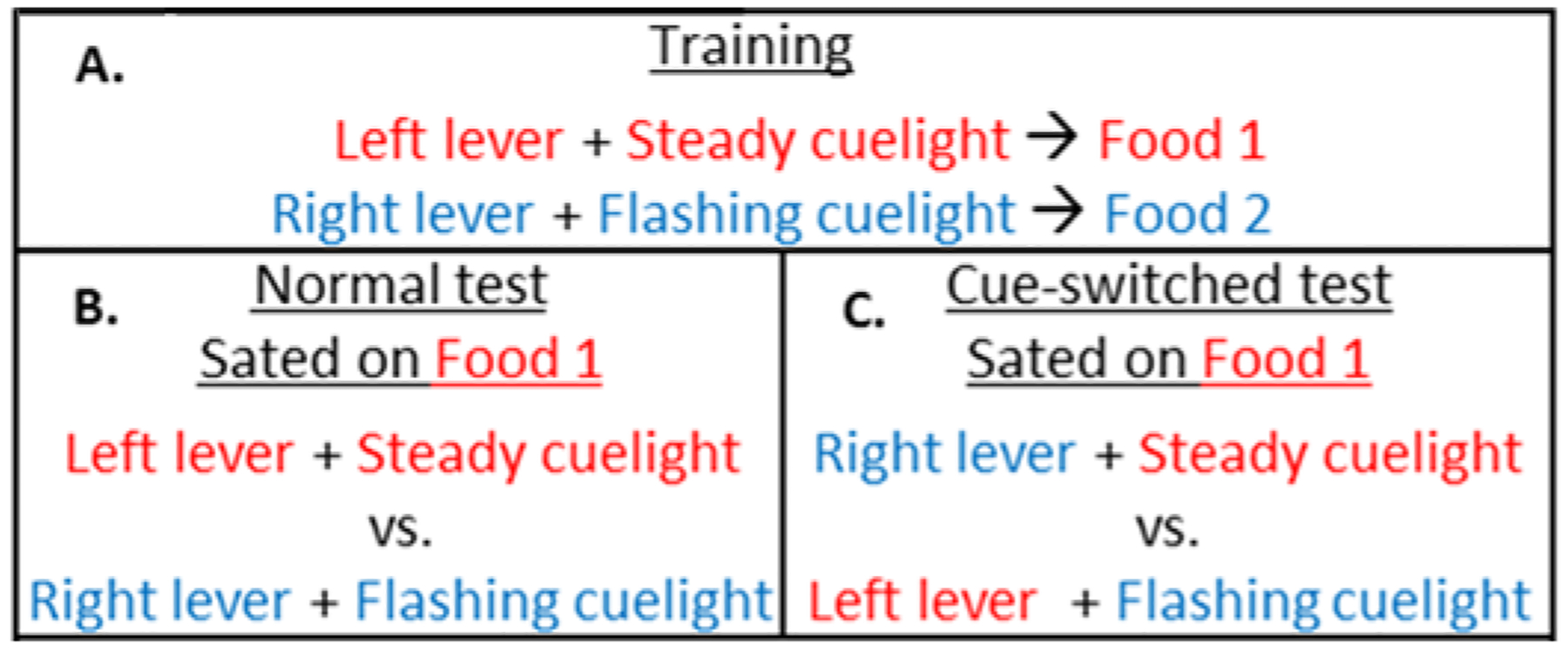
Training and testing procedures. (A) Cued-trial operant training. (B) Cue Normal test lever-light compounds. (C) Cue Switched test lever-light compounds

Our lab has recently begun to examine the possibility of compensation between strategies using a new version of the devaluation task. In our devaluation task, lever-light compounds earn separate reinforcers (e.g. left lever + steady cue-light earns precision pellets and right lever + flashing cue-light earns chocolate pellets) (Fig. 1A). Our previous research showed that pretraining inactivation of basolateral amygdala (BLA) or mediodorsal thalamus (MD), but not lateral orbitofrontal cortex (lOFC) or PL impaired goal-directed behavior in our task if the lever-light-food compounds are the same in testing as in training (a Cue Normal Test- in which lever-location and cue-lights give congruent information about outcomes) (Fig. 1B) [40]. Our finding that inactivation of lOFC or PL did not impair devaluation was unexpected, as the prior literature suggests that either lOFC or PL should be necessary for goal-directed behavior. If responses to a fixed location earn an outcome, goal-directed behavior is disrupted by pretraining PL inactivation/lesions [25–29]. In tasks where discrete cues (like lights or presentation of two response options during trials separated by an intertrial interval) indicate the expected outcome and/or time to respond, goal-directed behavior tends to be disrupted by lOFC inactivation/lesions [30–39, 41–43]. However, our inactivation results could be explained if the lever-location and discrete cues above the levers provide redundant outcome information, allowing the rats to use alternative strategies when the neural substrates supporting one strategy are nonfunctional. In support of this hypothesis, our preliminary research (available in preprint form) showed that devaluation behavior is impaired by pretraining combined lesions of both PL and lOFC (which would prevent compensation between the two strategies putatively supported by the two brain areas) but not lesions of either PL or lOFC alone [44]. Likewise, rats with pretraining PL lesions are impaired in devaluation in a test in which the lever-location and cue-lights are mismatched between training and testing (a Cue Switched Test- in which lever-location and cue-lights give incongruent information about outcomes and compensation between strategies cannot maintain similar behaviors- see Fig. 1C) (unpublished data).

In addition, most animal research on long-term effects of exposure to addictive drugs on behavior in the devaluation task examined effects of exposure during adulthood, but human drug use often begins during adolescence and the age of exposure is associated with differential outcomes. A substantial portion of the American population uses alcohol or illicit drugs during the teenage years [45]. For example, according to one recurring yearly survey, 50–70%, 30–40%, and 10–20% of American 12^th^ graders reported having used alcohol, marijuana/hashish, and illicit drugs other than marijuana/hashish, respectively, within the last year in each year from 2005–2020 [46]. Studies have found evidence that the majority of people admitted for treatment of substance use began this use prior to age 18 [47], that there is a linear decrease in the risk of developing a substance use problem within 7 years as the age of first use increases from 13–14 to 17 [48], and that initiation of use of alcohol or marijuana prior to age 14 leads to a greater than 4X increase in the rate of diagnosed substance use problems for that substance compared with people who initiated use after age 18 [49]. Previous research from rodent models also suggests that there may be more significant long-term behavioral and neurobiological effects of drug exposure if this exposure occurs during adolescence, rather than adulthood (as reviewed in [50]).

Here, we tested whether adolescent exposure to the psychostimulant amphetamine or adolescent exposure to the dissociative anesthetic drug ketamine would lead to impairment in goal-directed behavior in our task. We also tested whether any such deficits might be hidden by compensation in a version in which both strategies lead to the same behavioral pattern during testing. Prior research has demonstrated behavioral impairments weeks after repeated exposure to psychostimulants (including methamphetamine, amphetamine, and cocaine) in devaluation tasks requiring cue-based or lever-location strategies [51–56] (but see [57–59] for alternative explanations of these findings- detailed in the Discussion section). For Experiment 1, we based our amphetamine dosing regimen on a previous experiment which suggested that 3 mg/kg injections of amphetamine every-other day from post-natal day (PND) 27–45 led to decreased bursting in PL in response to saline injections during adulthood [60]. For Experiment 2, our dosing regimen was a modified version of the one used in previous experiments using repeated subanesthetic injections of 30 mg/kg ketamine. This dosing regimen has been shown to lead to behavioral impairments consistent with dysfunction of PL and/or lOFC (see [61] for results consistent with lOFC dysfunction based on previous research [62–64] and see [65] for results consistent with PL dysfunction based in previous research [63, 66]). However, we combined the twice daily injections from [61] with the 10 days of injections from [65]. Although the particular regimen differed between ketamine (twice daily injections daily for 10 days) and amphetamine (an injection every other day), both regimens finished on PND 44–45. To the best of our knowledge, this is one of the first (and possibly the first) examination of the long-term effects of adolescent psychostimulant or dissociative anesthetic exposure in the devaluation task. As described below, we found no evidence for impaired goal-directed behavior in either test type after either manipulation. We then discuss several factors that may determine whether exposure to drugs of abuse leads to long-term devaluation impairments.

## 2. Materials and Methods

### 2.1 Subjects

Long Evans rats were bred in our facility. For the Exp. 1 data, there were 50 rats (21 male, 29 female). For the Exp. 2. data, there were 103 rats (48 male, 55 female). Rats were weaned on PND 21. Following weaning, animals were individually housed and maintained on a 12-hour reverse light-dark cycle with lights off at 07:00 am in a temperature and humidity-controlled room. Water was available *ad libitum* throughout the experiment and food was available *ad libitum* until the beginning of the food restriction period. At PND66, the rats were food-restricted to 85% of their initial free-feeding weight by daily feedings with a minimum of 5 g of food chow per day. Once rats reached their 85% target weights, the target body weight increased by 1 g/day for males or 0.25 g/day for females for the remainder of the experiment, such that the rats’ target weights gradually increased by 7 g/week or 1.75 g/week. The different feeding regimens in male and female rats are meant to cause similar leverpress rates in the two sexes (determined from our lab’s pilot data), and is consistent with our published research using the devaluation task [40].

### 2.2 Animal ethics statement

All procedures and animal care were approved by and conducted in accordance with the Kansas State University Institutional Animal Care and Use Committee guidelines and were conducted in accordance with the U.S. National Institutes of Health Guidelines for the Care and Use of Laboratory Animals. All rats were euthanized with a lethal dose of Fatal-Plus (pentobarbital solution) followed by decapitation as a secondary method of confirming death after the completion of the experiments, consistent with the recommendations of the Panel on Euthanasia of the American Veterinary Medical Association.

### 2.3 Apparatus

The behavioral training occurred in 12 operant chambers (Med Associates, St Albans, VT). Each chamber had a pellet dispenser that was used to deliver 45-mg precision pellets (catalog #1811155; TestDiet, Richmond, IN), 45-mg chocolate-flavored sucrose pellets (catalog #1811256; TestDiet, Richmond, IN), or 45-mg grain pellets (catalog #1812127; TestDiet, Richmond, IN). The particular pellet that was delivered depended upon the phase of the experiment. Each chamber was equipped with two retractable levers on either side of the food-cup at approximately one-third of the chamber’s total height. A white cue-light was located above each of the levers. A red house-light was located on the back wall in the top–center. The equipment was controlled by (and all responses were recorded by) a Dell OptiPlex computer (Dell Inc., Round Rock, TX) with Med-PC for Windows (Med Associates, St Albans, VT).

### 2.4. General behavioral training and testing

Behavioral training occurred after rats had acclimated to the restricted feeding conditions. This training occurred during the dark phase of the light:dark cycle. Rats were weighed and received their daily feeding after each day’s training session.

This experiment utilized the same training and testing procedures used and described in detail by Fisher, Pajser, and Pickens [40]. In brief, the rats were trained with three magazine training sessions (one with each of the three types of food pellets- chocolate, grain, and precision). The magazine training sessions occurred once-daily for 40 min. During each session, one of the pellets was delivered every 125 seconds. The rats were then trained to leverpress in two fixed-interval-1 training sessions during each day for two days (with afternoon sessions occurring 2–4 hours after the morning sessions). In these sessions, rats could earn up to 50 grain pellets and leverpresses could earn a pellet each second under the fixed-interval-1 schedule. Each lever was trained in a separate session for a maximum of 50 pellets or 60 minutes per session, with training for each lever in the morning on one day and in the afternoon on the other day.

After this pretraining was complete, the rats were trained in 4 cued-trial operant sessions, given once daily. The rats were trained with two sessions for each of the two lever-cue-reinforcer relationship. During the first and the fourth training session, responses to the left lever (with illumination of a steady white cue-light above the lever) resulted in precision pellets. During the second and the third training session, responses to the right lever (with flashing [2 Hz] illumination of white cue-light above it) resulted in chocolate pellets. Each cued-trial training session lasted for 40 min and contained 40 trials. During each 40-sec trial, leverpresses could earn two pellets, with one pellet available at a variable time during the first 20 sec and pellet available at a variable time during the last 20 sec (a modified VI20 reinforcement schedule during the trials). Rats could earn a maximum of 80 pellets during each session. During the inter-trial intervals (13–19 sec with a mean of 20 sec), the levers were retracted without any illumination of the cue-lights.

Following cued trial training, a choice test was administered. In the operant chambers, rats received a 1-h satiation session with access to 30 g of either chocolate or precision pellets (identity counterbalanced) presented in ceramic bowls. Fifteen minutes after the satiation period, rats received a 12-min choice test with twelve 40-sec trials. During each trial, both levers with cue-lights were presented and responding was measured. In the Cue Normal condition, the cue-lights above the levers were in the same position as during training (e.g.: steady cue-light above the left lever). Responding should be decreased for the cue-light+lever compound that earns the devalued reinforcer if rats devalue properly. For the Cue Switched condition, the cue-lights above the levers were in the opposite position as during training, such that the flashing light was above the left lever and the steady light was above the right lever. Our previous research suggests that neurobiologically intact rats decrease responses made to the lever in the fixed location that previously earned the devalued/sated food rather than the lever below the cue-light associated with the sated food under these conditions [40]. However, our preliminary research suggests that PL lesions lead to impaired devaluation with a trend towards lower responding to the lever below the cue-light associated with the sated food, even though this lever-location is associated with the non-sated food (unpublished data). Lever presses did not earn pellets during the test to ensure the rats used their memory representations of the cue-lever combinations to guide behavior. Next, rats received two cued-trial retraining sessions, one with each lever+cue-light-food compound (with the original configurations used during training), to return lever pressing rates to baseline. Rats then completed another choice test with the opposite pellet devalued during the satiation period and with the same cue-light-+lever combinations as in the first test (so Cue Switched groups once again had mismatched cue-light+lever compounds between training and test). Testing with both pellets sated controls for any differences in preference for them, as we often observe higher lever-responding for the precision pellets and higher consumption of them during the satiation period (see [67] for an example). Any preference should be averaged out by testing with both pellets sated. No rats were excluded for low responding in the choice tests. However, in Experiment 1, one rat was excluded because of a jammed feeder that led to extinction of the lever-press response and one rat was excluded as an experimental error led to them not being tested in a second choice test.

### 2.4 Specific Experiments

#### 2.4.1 Experiment 1- Effects of repeated amphetamine injections during adolescence

Fifty-one rats received 10 intraperitoneal (i.p) injections of either amphetamine (3 mg/kg, Sigma-Aldrich) or an equivalent volume of sterile saline (1 ml/kg) every other day from PND 27–45. After the final injection, rats were kept in their home cages with *ad libitum* access to food and water until food restriction began at PND 66. The rats then received training and testing as described above. The number of rats in each drug- and testing-condition was: Amphetamine-Cue Normal = 6 males and 8 females, Amphetamine-Cue Switched = 5 males and 7 females, Saline- Cue Normal = 5 males and 8 females, Saline-Cue Switched = 5 males and 6 females.

#### 2.4.2 Experiment 2- Effects of repeated ketamine injections during adolescence

One-hundred and three rats received twice daily i.p. injections of either 30 mg/kg ketamine or 2 ml/kg sterile saline solution from PND35–44 (20 injections total). After the final injection, rats were kept in their home cages with *ad libitum* access to food and water until food restriction began at PND 66. The rats then received training and testing as described above. The number of rats in each drug- and testing-condition was: Ketamine-Cue Normal = 12 males and 12 females, Ketamine-Cue Switched = 12 males and 15 females, Saline-Cue Normal = 12 males and 11 females, Saline-Cue Switched = 12 males and 17 females.

## 3. Results

### 3.1 Experiment 1

#### 3.1.1 Cued-trial lever training results

**Fig. 2.**
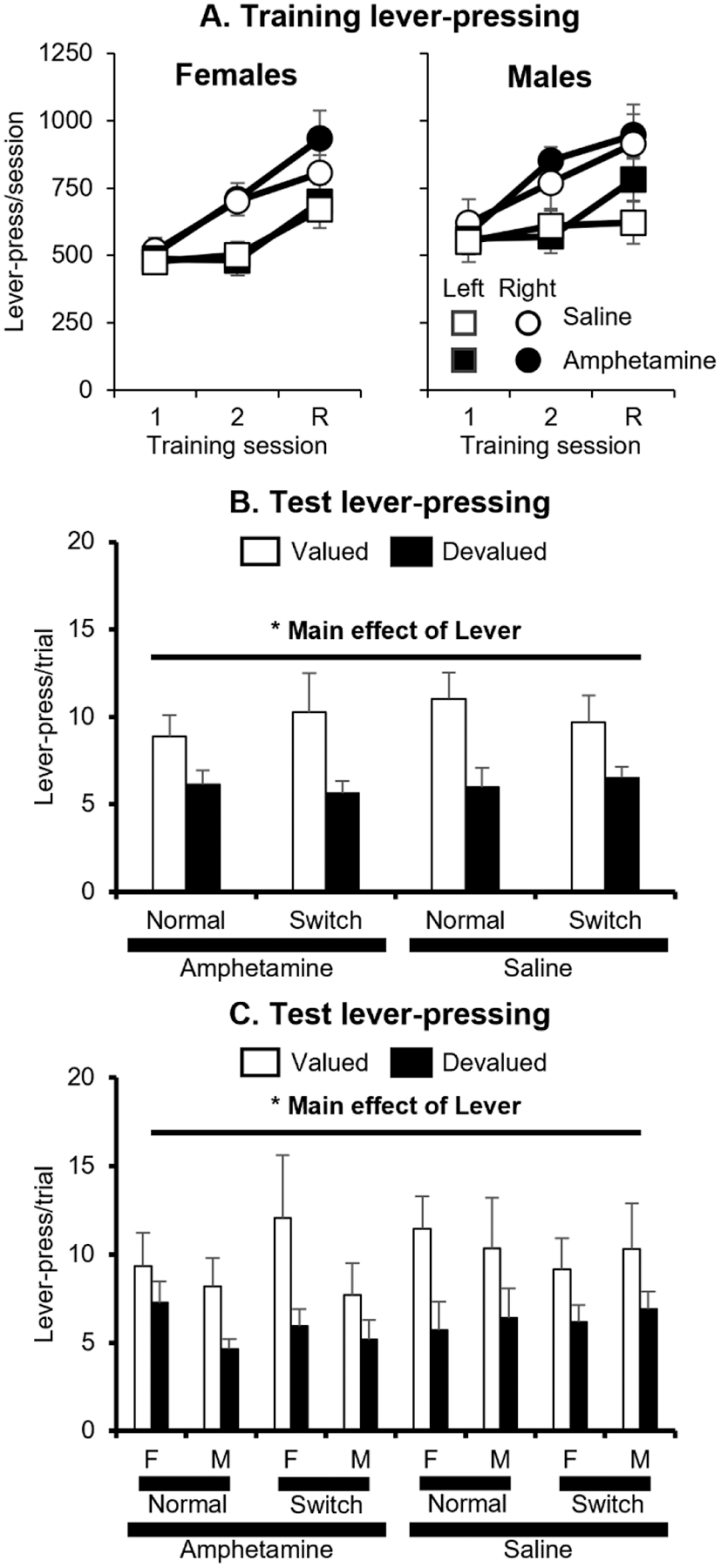
Experiment 1 data. (A) Leverpresses/session (mean + SEM) during training. Left = female groups. Right = male groups. Circles = pressing on left lever for precision pellets. Squares = pressing on right lever for chocolate pellets. Black symbols = amphetamine groups. White symbols = saline groups. On x-axis, 1 and 2 represent first 2 pre-test training sessions for each pellet. R represents the retraining session for each pellet between the two tests. (B) Lever presses/trial (mean ± SEM) in each group during the devaluation test in Exp. 1 (averaged test). (C) White bars represent responding on the nondevalued lever. Black bars represent responding on the devalued lever. F = female. M = male. Normal = behavior in the Cue Normal test. Switch = behavior in the Cue Switched test

We analyzed the number of leverpresses/session in the training phase (Fig. 2A) with a mixed-factor ANOVA with the between-subjects factors of Treatment (Amphetamine and Saline), Sex (Male and Female), and Test Type (Cue Normal and Cue Switched; this represented the future test condition after training) and the within-subjects factors of Pellet/Lever (Precision/Left vs. Chocolate/Right) and Training Day. This analysis found significant effects of Pellet/Lever *F*(1, 42) = 34.4, *p* < 0.0001 and Training Day *F*(2, 84) = 45.3, *p* < 0.0001 and a significant interaction of Pellet/Lever X Training Day *F*(2, 84) = 14.9, *p* < 0.0001. No other main effects or interactions were significant (all *p* > 0.05). A post-hoc analysis of the Pellet/Lever X Training Day interaction revealed that rats made more presses/session for the left lever that earned precision pellets than for the right lever that earned chocolate pellets on the second training day and during the retraining session between the tests (both *p* < 0.01), but not in the first training day for each pellet (*p* > 0.10).

#### 3.1.2 Choice test results

We analyzed the test results (Fig. 2B,C) using a mixed-factor ANOVA with the between-subjects factors of Treatment (Amphetamine and Saline), Sex (Male and Female), and Test Type (Cue Normal and Cue Switched) and the within-subjects factor of Lever (Devalued and Nondevalued). This analysis found a significant effect of Lever *F*(1, 42) = 25.9, *p* < 0.0001. No other main effects or interactions were significant (all *p* > 0.22). These results indicate that all groups showed sensitivity to outcome devaluation regardless of whether they had a history of drug or saline exposure.

### 3.2 Experiment 2

#### 3.2.1 Cued-trial lever training results

**Fig. 3.**
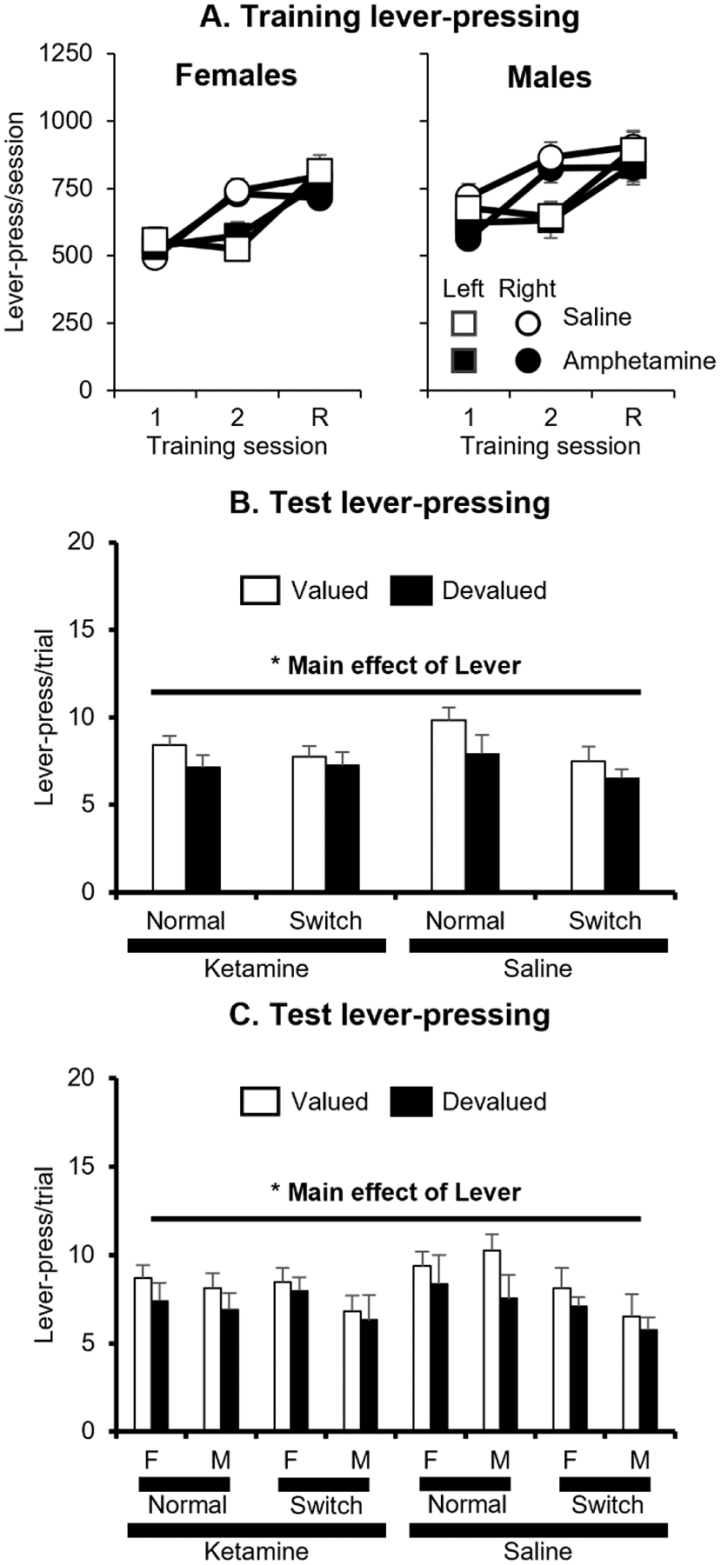
Experiment 2 data. (A) Leverpresses/session (mean + SEM) during training. Left = female groups. Right = male groups. Circles = pressing on left lever for precision pellets. Squares = pressing on right lever for chocolate pellets. Black symbols = ketamine groups. White symbols = saline groups. On x-axis, 1 and 2 represent first 2 pre-test training sessions for each pellet. R represents the retraining session for each pellet between the two tests. (B) Lever presses/trial (mean ± SEM) in each group during the devaluation test in Exp. 1 (averaged test). (C) White bars represent responding on the nondevalued lever. Black bars represent responding on the devalued lever. F = female. M = male. Normal = behavior in the Cue Normal test. Switch = behavior in the Cue Switched test.

We analyzed the number of leverpresses/session during training (Fig. 3A) using a mixed-factor ANOVA with the between-subjects factors of Treatment (Ketamine and Saline) and Sex (Male and Female) and the within-subjects factors of Pellet/Lever (Precision/Left and Chocolate/Right) and Training Day. This analysis found significant effects of Sex F(1, 99) = 6.6, *p* = 0.01, Pellet/Lever F(1, 99) = 8.4, *p* = 0.005 and Training Day F(2, 198) = 98.7, *p* < 0.0001 and a significant interaction of Pellet/Lever X Training Day F(2, 198) = 43.0, *p* < 0.0001. No other main effects or interactions were significant (all p > 0.15). A post-hoc analysis of the Pellet/Lever X Training Day interaction revealed that rats made more presses/session for the left lever that earned precision pellets than for the right lever that earned chocolate pellets on the second training day (*p* < 0.01), but not in the first training day for each pellet or the retraining session between the tests (all *p* > 0.10).

#### 3.2.2 Choice test results

We analyzed the test results (Fig. 3B,C) using a mixed-factor ANOVA with the between-subjects factors of Treatment (Ketamine and Saline), Sex (Male and Female), and Test Type (Cue Normal and Cue Switched) and the within-subjects factor of Lever (Devalued and Nondevalued). This analysis found significant effects of Test Type *F*(1, 95) = 4.8, *p* = 0.03 and Lever *F*(1, 95) = 5.7, *p* = 0.02. No other main effects or interactions were significant (all *p* > 0.05). These results indicate that all groups showed sensitivity to outcome devaluation regardless of whether they had a history of drug or saline exposure, although the overall devaluation effect was fairly weak even in the Saline rats. Visual inspection of the devaluation effect in Experiment 2 suggested that the devaluation effect may have been smaller in the Cue Switched condition, but this was not supported by a Lever X Test Type interaction.

### 3.3 Preference ratios

We have traditionally assessed devaluation effects in our previous research by assessing the number of leverpresses, but an alternative measure of the devaluation effect can be calculated by assessing the ratio of responding on the lever that previously earned the nondevalued food to responding on the lever that previously earned the devalued food. We calculated this using the formula (with the “nondevalued” and “devalued” lever designated based on the levers’ fixed spatial location):

$$\text{Preference ratio}=\frac{\left(\text{Responses on the nondevalued lever }-\text{ Responses on the devalued lever}\right)}{\left(\text{Responses on the nondevalued lever }+\text{ responses on the devalued lever}\right)}$$

This measure normalizes the change in responding based on the overall level of responding. For example, using this measure, a low responding rat that presses 4 times on the nondevalued lever and 2 times on the devalued lever would have a preference ratio equivalent to another rat that presses 30 times on the nondevalued lever and 15 times on the nondevalued lever. In this case, exclusive responding on the nondevalued lever results in a preference ratio of 1.0, exclusive responding on the devalued lever results in a preference ratio of −1.0, and equal responding on the 2 levers results in a preference ratio of 0.0.

#### 3.3.1 Preference ratios in Experiment 1

**Fig. 4.**
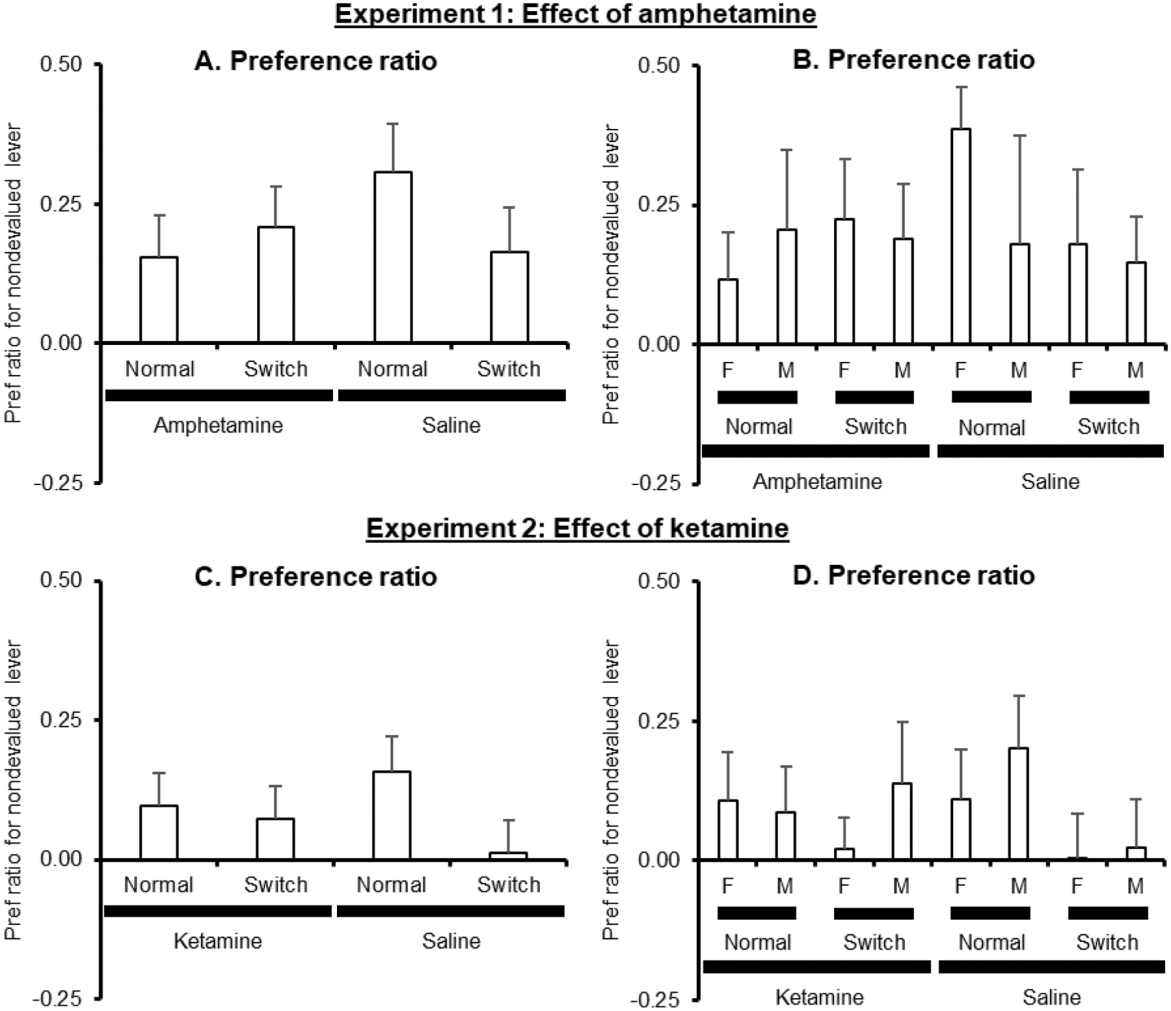
Preference ratio data (mean + SEM) in each group during the devaluation test from Experiments 1 and 2. (A) Preference ratio in Experiment 1 averaging across sex. (B) Preference ratio in Experiment 1 separated by sex. (C) Preference ratio in Experiment 2 averaging across sex. (D) Preference ratio in Experiment 2 separated by sex. Pref = preference. F = female. M = male. Normal = behavior in the Cue Normal test. Switch = behavior in the Cue Switched test

We analyzed the test results of Experiment 1 (Fig. 4A,B) using a mixed-factor ANOVA with the between-subjects factors of Treatment (Amphetamine and Saline), Sex (Male and Female), and Test Type (Cue Normal and Cue Switched). This analysis found a no significant main effects or interactions (all *p* > 0.32). We then examined whether the overall pattern using this measure supported a conclusion that goal-directed action was being expressed using a single-sample t-test. As there were no differences between groups, we compared the preference ratio across the entire sample to 0 (the score that would indicated that there was no preference). We found that the preference score for the sample was significantly different from 0: *t*(46) = 5.3, *p* < 0.001. These results indicate that there was a significant devaluation effect (based on the lever-location) in Experiment 1 when the scores were normalized for the overall level of responding and that there was no evidence that the magnitude of this devaluation effect using this measure differed across the different groups.

#### 3.3.1 Preference ratios in Experiment 2

We analyzed the test results of Experiment 2 (Fig. 4C,D) using a mixed-factor ANOVA with the between-subjects factors of Treatment (Ketamine and Saline), Sex (Male and Female), and Test Type (Cue Normal and Cue Switched). This analysis found a no significant main effects or interactions (all *p* > 0.19). We then examined whether the overall pattern using this measure supported a conclusion that goal-directed action was being expressed using a single-sample t-test. As there were no differences between groups, we compared the preference ratio across the entire sample to 0 (the score that would indicated that there was no preference). We found that preference score for the sample was significantly different from 0: *t*(102) = 2.7, *p* < 0.01. These results indicate that there was a significant devaluation effect (based on the lever-location) in Experiment 2 when the scores were normalized for the overall level of responding and that there was no evidence that the magnitude of this devaluation effect using this measure differed across the different groups.

## 4. Discussion

Our results provided no evidence for impairment of goal-directed behavior in a devaluation task after adolescent amphetamine or ketamine injections. This was true regardless of whether we used a test in which cue-light and lever-location information were congruent between training and test (allowing for compensation between strategies supported by different frontal cortex areas) or incongruent between training and test (preventing compensation between strategies). Below, we discuss several possible reasons for this lack of these effects of drug exposure and discuss the information our results can and cannot provide about the strategies used to guide devaluation in our task.

### 4.1 Evidence for primary use of a lever-location strategy to guide behavior in our task with conflicting information

Our results were not informative about the possibility of compensation between strategies after drug exposure. We designed and conducted the experiments specifically to examine the possibility of impaired devaluation in the Cue Switched condition and not in the Cue Normal condition, which would indicate compensation between strategies used to guide goal-directed behavior. The failure to find impaired devaluation in the Cue Switched condition in the drug-exposed groups means our results are uninformative about whether compensation after drug exposure can help to maintain goal-directed behavior. However, our results largely replicate our previous findings about the relative role of the two types of strategies that can guide behavior. In both experiments, we replicated our previous pattern in which rats tested under the Cue Switched conditions primarily guide their responding according to the fixed location of the lever rather than based on the cue-light’s location [40]. Visual inspection of the devaluation effect in Experiment 2 using the leverpress/trial measure (Fig. 3B,C) suggested that the devaluation effect may have been smaller in the Cue Switched condition, but this was not supported by a Lever X Test Type interaction in the analysis of either the leverpress/trial measure or the preference ratio measure in Experiment 2. This visual pattern was not observed in Experiment 1. Additional research is necessary to assess whether different training conditions could lead to goal-directed behavior that is guided by cue-lights in neurobiologically intact rats.

### 4.2 Relation of our null effect to prior literature on long-term behavioral effects of psychostimulant exposure on lOFC- and PL-dependent tasks

Our finding that amphetamine exposure did not impair goal-directed behavior contrasts with several prior demonstrations that psychostimulant exposure can lead to altered PL and/or lOFC function and goal-directed behavior impairments in devaluation tasks dependent on PL or lOFC function. Prior research has shown that psychostimulant exposure (through forced exposure or self-administration) alters the activity of PL and/or lOFC. For example, repeated amphetamine/cocaine injections or methamphetamine self-administration increases excitability and/or firing rate in PL days or weeks later [68–72]. Previous cocaine exposure also disrupts selective lOFC firing to cues that predict specific outcomes in a discrimination task [73]. There is also evidence for impairments in goal-directed behavior weeks after the final cocaine exposure in a devaluation task requiring a cue-based strategy [56], although the impairment could be explained by training and testing in a drug-paired environment rather than neurotoxic dysfunction [58, 59]. Likewise, prior research has demonstrated behavioral impairments weeks after the final cocaine, amphetamine, or methamphetamine exposure in a devaluation task requiring a lever-location strategy [51–55], although the impairment may be caused by faster habit learning rather than a failure of the neurobiological substrates of goal-directed behavior [57].

Our results may be congruent with the prior literature showing goal-directed behavior impairments in the devaluation task after prior psychostimulant exposure if these prior impairments were due to accelerated habit-formation (in tasks prone to formation of habits after extended training) or training/testing in drug-paired contexts rather than a stable neurotoxicity-induced dysfunction of the brain areas needed for goal-directed behavior. As noted above, prior research has demonstrated impairments in goal-directed behavior weeks after the final cocaine exposure in devaluation tasks requiring a cue-based strategy [56], but this experiment paired the behavioral training and testing environment with cocaine. Previous research has also shown that behavioral contexts associated with the psychostimulant methamphetamine lead to devaluation impairments that are not observed in the same rats when tested in a different context [58], so the cocaine-induced impairment in the cue-based task may be due to an interference effect from the cocaine-paired training and testing context. Many of the other demonstrations of impaired goal-directed behavior in devaluation tasks where behavior was guided by lever-location after cocaine or amphetamine exposure have used task versions with training with one lever-reinforcer relationship [51, 52, 54, 55] or a second response-reinforcer relationship was trained only after the cessation of training for the other response-reinforcer relationship [53]. Extended training can lead to habit-based devaluation insensitivity with variable interval schedules and/or extended training if only one response-reinforcer relationship is trained or if response-reinforcer relationships are trained in different contexts [1, 27], but goal-directed behavior in operant responding in rodents occurs if multiple response-reinforcer relationships are trained in the same context [74].

Our results mirror a pattern observed in multiple-response/multiple-reinforcer operant devaluation (conditions that don’t tend to lead to habit-formation) after exposure to other drugs of abuse. Prior exposure to subchronic cocaine or methamphetamine [57, 58, 75] or a neurotoxic single-day regimen of methamphetamine [76] do not cause a generalized goal-directed behavior impairment in devaluation tasks where two different response options lead to separate rewards, possibly because these procedures are resistant to the formation of habits [74]. In these cases, cues associated with a drug can specifically interfere with goal-directed behavior in the presence of those cues [58, 75], but no impairment is seen when these cues are absent.

However, there are several methodological considerations. *First*, the timing of the amphetamine exposure may have affected our results. While previous research suggests that there may be more significant long-term behavioral and neurobiological effects of drug exposure if this exposure occurs during adolescence rather than adulthood (as reviewed in [50]), it is possible that amphetamine exposure during adolescence had less of a long-term effect on behavior than if it were given during adulthood. Relatedly, it is possible that we could have found an impairment in devaluation if we had trained and tested the rats with a shorter interval between amphetamine exposure and training/testing. *Second*, it is possible that higher doses of amphetamine could lead to impaired devaluation. We based our injection regimen and time of exposure on a previous study that showed that 3 mg/kg injections of amphetamine every-other day from PND 27–45 led to decreased bursting in PL in response to saline injections during adulthood [60]. Our 3 mg/kg dose was also higher than the 2 mg/kg dose that was previously shown to impair devaluation in a task that predisposes rats towards habit learning if rats are trained for enough sessions (in a previous experiment with 7 once daily injections for 7 days during adulthood) [54]. However, we did not assess the effects of amphetamine exposure at a range of doses and it is possible that higher doses would lead to devaluation impairments in tasks that do not predispose rats to habit learning if they are extensively trained. *Third*, while our predicted impairments in goal-directed behavior did not depend upon the presence of sensitization to the effects of psychostimulants, several of the previous reports of psychostimulant exposure impairing devaluation also showed that the impaired rats exhibited locomotor sensitization to the effect of the psychostimulant [54, 56]. As we did not assess locomotor sensitization, it is unclear whether our amphetamine exposure regimen led to sensitization. However, the level of sensitization did not correlate with the level of devaluation impairment in the previous experiments that assessed it [54, 56], so it is also unclear whether psychomotor sensitization is required for devaluation impairments. Finally, it is possible that the timing of our injections (specifically- during the dark phase of the light-dark cycle) may have affected our results, as there is prior evidence for the light-dark cycle affecting the sensitivity to, pharmacokinetics of, and/or intake of a variety of drugs of abuse (for example, see [77–80]). We gave injections during the dark phase to rats on a reverse light-dark cycle, while most of the previous experiment examining long-term effects of psychostimulants on devaluation and that reported their lighting conditions reported maintaining rats on a regular light cycle (with injections presumably occurring during the day) or specified injecting during the light phase [53, 54, 56, 58, 81] (but see [55]).

While we found relatively clear evidence that the particular amphetamine exposure regimen and washout period did not lead to devaluation impairment in our task, additional research is needed. Specifically, future research should 1) determine if our amphetamine exposure regimen would lead to accelerated habit formation in tasks that predispose rats towards habit learning if rats are extensively trained, 2) determine if this exposure regimen would impair behavior in the devaluation if given during adulthood, 3) determine if other exposure regimens such as higher doses or shorter washout periods might lead to a goal-directed behavior impairment, 4) determine if our amphetamine exposure led to an impairment in lOFC function that was obscured by intact PL function or whether it did not impair PL or lOFC function, and 5) determine if this exposure regimen leads to locomotor sensitization.

### 4.3 Relation of our null effect to prior literature on long-term behavioral effects of dissociative anesthetic exposure on lOFC- and PL-dependent tasks

Our finding that ketamine exposure did not impair goal-directed behavior contrasts with several prior demonstrations that dissociative anesthetic exposure can lead to altered PL and/or lOFC function and impairments in tasks dependent on PL or lOFC function, although the prior literature on devaluation tasks is relatively sparse. One previous experiment found that acute injections of the non-competitive NMDA receptor MK-801 between the satiation period and the leverpress devaluation test impaired the selective decrease in leverpressing for the reward in a devaluation task in which only one response-reward relationship had been trained (with decreased leverpressing regardless of whether the operant reinforcer or another food was sated) [82]. However, this experiment examined acute effects with pretest injections rather than long-term effects in a drug-free state. In addition, the NMDA receptor antagonist used (MK-801 vs. ketamine) and the type of devaluation task (single lever-reward free operant task vs. two response-cue-reward relationships) differed from the parameters used in our study. Therefore, it is unclear which factor or factors distinguish our results from this previous one. Conversely, our lab previously examined long-term effects of a ketamine regimen with higher doses and fewer injections (3 once-daily injections of a higher sub-anesthetic 50 mg/kg or anesthetic 100 mg/kg dose) and found no effects of ketamine exposure on goal-directed behavior in the same task used here with a Cue Normal test [83]. However, there was no Cue Switched test given, so it is unclear whether this represents intact function of PL, lOFC, or both, as goal-directed behavior in the Cue Normal task is intact if only PL or only lOFC is lesioned [44], and the fewer number of exposures may have led to a less pronounced effect than long-term exposure. Therefore, there is little to no data on what would be expected for long-term effects of our ketamine exposure regimen in the current experiment.

It is unclear whether our ketamine exposure regimen failed to alter PFC function or whether any impairments may have occurred but did not last until our training/testing period. Prior literature suggests that there are impairments in the reversal learning task (which is dependent on lOFC [62–64]) after subchronic exposure to ketamine or the NMDA receptor antagonist drug phencyclidine (PCP) under some conditions [61, 84–93] and no impairment under other conditions [65, 94–100]. Likewise, prior literature suggests that there are impairments in extradimensional shifting in the attentional set-shifting or strategy set-shifting tasks (which is dependent on PL [66, 101, 102]) after subchronic exposure to PCP or ketamine [61, 65, 85, 91, 95, 97–100]. There were relatively uniform regimens of exposure and washout in these previous studies, with the vast majority of experiments giving dissociative anesthetics during adulthood and having testing between 3 and 14 days later (compared with the much wider range in age of exposure and washout periods ranging from 24 h to 6 weeks for psychostimulant exposure and testing). While this simplifies the prior literature, it may limit the generalizability of these findings to make predictions about our experiment.

There are several methodological considerations for the interpretation of our findings. *First*, our devaluation effect was smaller in Experiment 2 than in Experiment 1. Although all 8 groups (across each Male vs. Female X Ketamine vs. Saline X Cue Normal vs. Cue Switched condition) exhibited numerically lower responding to the devalued lever than to the nondevalued lever, it is possible that this weaker overall devaluation effect may have made it more difficult to detect any potential decrease in the magnitude of the devaluation effect after ketamine. This was particularly evident in visual inspection of Saline group rats given Cue Switched conditions, although there was no significant Test Type X Lever interaction. *Second*, it is possible that higher doses of ketamine could lead to impaired devaluation. We based our injection regimen on dosing regimens previously shown to lead to behavioral impairments consistent with dysfunction of PL and/or lOFC [61, 65]. However, we did not assess the effects of ketamine exposure at a range of doses or lengths of exposure, and it is possible that higher doses or more extensive exposure are required to lead to devaluation impairments. *Third*, the timing of the ketamine exposure may have affected our results. As the experiments reviewed in the previous paragraph were performed on rodents during or near adulthood and they all began behavioral testing within ~2 weeks or less after the final PCP or ketamine injection, it is unclear how relevant they are for predicting PL and lOFC function more than 5 weeks after ketamine exposure in adolescent rats. While previous research suggests that there may be more significant long-term behavioral and neurobiological effects of drug exposure if this exposure occurs during adolescence, rather than adulthood (as reviewed in [50]), it is possible that ketamine exposure during adolescence had less of a long-term effect on behavior than if it were given during adulthood. In addition, previous research has found that an identical once-daily PCP exposure led to opposite effects on activity regulated cytoskeletal-associated protein (Arc) mRNA in PL and lOFC 3 days after the final injection in rats of different ages, with decreased mRNA in adolescent rats and increased mRNA if given during early adulthood [103]. Alternatively, it is possible that adolescent ketamine exposure *did* lead to altered function of PL and/or lOFC for several weeks after exposure, but the more extended washout period (more than 5 weeks from the final injection until cued-trial training began) in the current experiment led to a recovery over time.

While we found no evidence that the particular ketamine exposure regimen and washout period led to devaluation impairment in our task, additional research is needed. Specifically, future research should 1) determine if our ketamine exposure regimen would lead to accelerated habit formation in tasks that predispose rats towards habit learning if rats are extensively trained, 2) determine if this exposure regimen would impair behavior in the devaluation if given during adulthood, 3) determine if other exposure regimens such as higher doses or shorter washout periods might lead to a goal-directed behavior impairment, and 4) determine if our ketamine exposure regimen led to an impairment in lOFC function that was obscured by intact PL function or whether it did not impair PL or lOFC function.

## 5. Conclusions

In conclusion, our results provided no evidence for impairment of goal-directed behavior after adolescent amphetamine or ketamine injections, regardless of whether we used a devaluation test that allowed for compensation between strategies or a test that prevented compensation between strategies. It is unclear whether this represents 1) a lack of effect of prior drug exposure (at this particular dosing regimen, age of exposure, and washout period) on the neural substrates of goal-directed behavior in tasks that do not predispose animals towards habitual behavior and do not present drug-related contextual cues during training/testing, 2) neurobiological dysfunction that faded over our longer washout period, or 3) impaired lOFC function obscured by intact goal-directed behavior based on the lever-location. Additional research is needed to differentiate between these possibilities.

## Data Availability

Data will be provided upon request to any qualified person who requests it.
